# Fetal Position Manifestation in Acute Focal Bacterial Nephritis

**DOI:** 10.31662/jmaj.2024-0091

**Published:** 2024-08-09

**Authors:** Junki Mizumoto

**Affiliations:** 1 Department of Family Practice, Ehime Seikyo Hospital, Ehime, Japan; 2 Center for General Medicine Education, School of Medicine, Keio University, Tokyo, Japan

**Keywords:** acute focal bacterial nephritis, fetal position, urinary tract infection

A 25-year-old woman presented with left flank pain and fever for 5 days. Two days before the presentation, the patient visited another hospital because of a fever and was prescribed amoxicillin plus clavulanic acid (2,000 and 500 mg/d), with no culture test performed. Despite the antibiotic treatment, the symptoms worsened, prompting the patient’s visit to our hospital.

The patient sat in a wheelchair with the hip joint flexed outward, knee joint flexed, and back bent forward. Physical examination revealed tapping pain on the left costovertebral area and a positive psoas sign. A contrast computed tomography scan confirmed multiple areas of decreased enhancement in the left kidney (Figure 1), which was consistent with acute focal bacterial nephritis (AFBN). The patient had no predisposing factors. Blood and urine cultures were negative. Administering cefmetazole 3 g/d rapidly improved the resolution of fever and flank pain.

**Figure 1.**
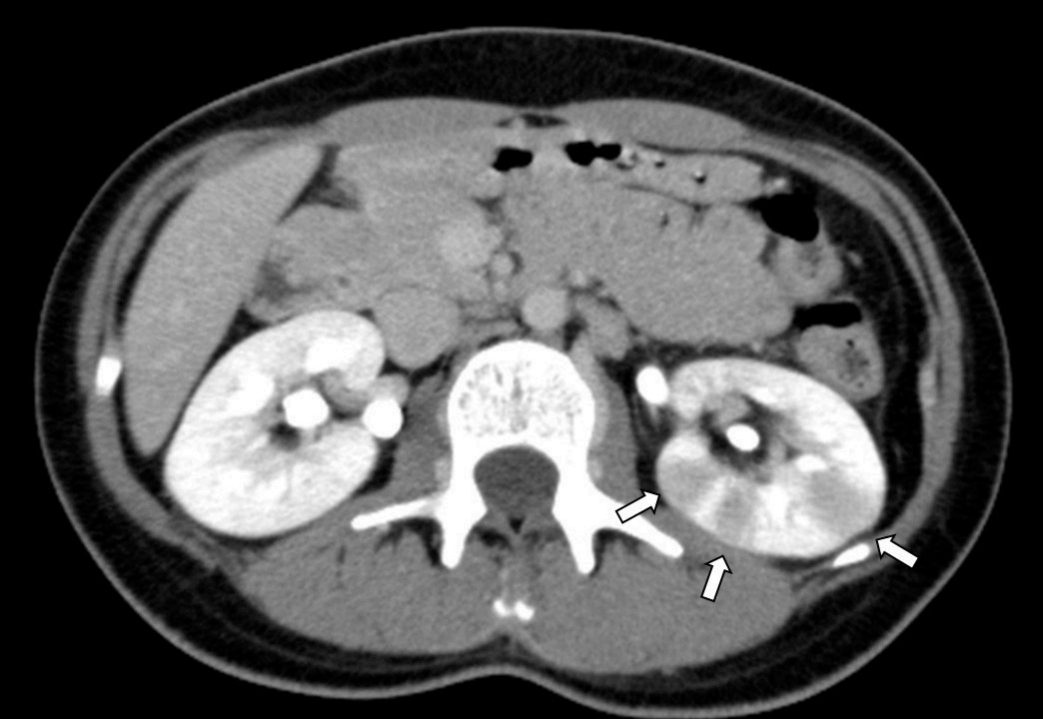
Multiple areas of decreased enhancement in the left kidney (white arrow).

Flank pain is a common symptom in AFBN ^(1)^; however, it cannot independently establish a diagnosis ^(2)^. The patient assumed a fetal position, characterized by leaning forward and curling up in the knee-to-chest posture, which is also observed in patients with acute pancreatitis, aiming to reduce the stretch on the pancreas ^(3)^, as well as in patients with psoas abscess, intending to alleviate strain on the psoas muscle ^(4)^. Herein, the patient presumably adopted the fetal position to potentially mitigate additional tension on the Gerota fascia.

Visual inspection―a physical examination―is often omitted ^(5)^. The present report conveys a strong message: a hawk-eyed physician can notice the patient’s characteristic posture early in the patient encounter and provide a prompt diagnosis. Fetal position suggests retroperitoneal inflammation, including AFBN with tensive Gerota fascia, and be crucial to prevent progression to renal abscess ^(6)^.

## Article Information

### Conflicts of Interest

None

### Author Contributions

Concept: J.M.; Design: J.M.; Data Collection and Processing: J.M.; Analysis and Interpretation: J.M.; Literature Search: J.M.; Writing: J.M.

### ORCID iD

Junki Mizumoto: 0000-0002-0783-7351

### Informed Consent

Informed consent was obtained from the patient.
